# TGF-β and IL-10 Production by HIV-Specific CD8+ T Cells Is Regulated by CTLA-4 Signaling on CD4+ T Cells

**DOI:** 10.1371/journal.pone.0008194

**Published:** 2009-12-14

**Authors:** Mohamed Elrefaei, Candace M. Burke, Chris A. R. Baker, Norman G. Jones, Stephanie Bousheri, David R. Bangsberg, Huyen Cao

**Affiliations:** 1 California Department of Public Health, Richmond, California, United States of America; 2 Harvard Initiative for Global Health, Massachusetts General Hospital, Cambridge, Massachusetts, United States of America; Beijing Institute of Infectious Diseases, China

## Abstract

Immune dysregulation in HIV-1 infection is associated with increased expression of inhibitory molecules such as CTLA-4, TGF-β, and IL-10. In this study we examined one potential mechanism for regulating TGF-β and IL-10 expression by HIV-specific suppressor CD8+ T cells. No overlap between TGF-β, IL-10, and IFN-γ cytokine production by HIV-specific CD8+ T cells was observed. TGF-β positive and IL-10 positive cells were FOXP3 negative, CD25 negative, and displayed a heterogeneous surface expression of CD127. TGF-β and IL-10 positive CD8+ T cells did not express CTLA-4. Nevertheless, CTLA-4 blockade resulted in a significant decrease in HIV-specific TGF-β positive and IL-10 positive CD8+ T cell responses, and a concomitant increase in HIV-specific IFN-γ positive CD8+ T cell responses. Depletion of CD4+ T cells abrogated the impact of CTLA-4 on HIV-specific TGF-β positive and IL-10 positive CD8+ T cells. Our study suggests that CTLA-4 Signaling on CD4+ T cells regulates the inhibitory functions of the HIV-specific suppressor CD8+ T cells.

## Introduction

Suppressor CD8+ T cells (CD8+ Ts) exert their inhibitory effects on T cell proliferation and cytokine production through a cell-cell contact-dependent mechanism [1]–[4], and by secreting the immunosuppressive cytokines interleukin-10 and TGF-β [5], [6]. CD8+ Ts mediate non-antigen specific suppression of T cell responses via IL-10 [7]–[10]. These suppressor CD8+ T cells are typically CD28 negative [11], [12] and express CD25, FOXP3, and CTLA-4 [1], [4], [13], [14]. Transforming growth factor β (TGF-β) is another immunosuppressive cytokine that inhibits T cell functions [15]–[18]. TGF-β up-regulates CTLA-4 expression [19]–[21] and suppresses T cell proliferation and IL-2 production [22]. Increased TGF-β and IL-10 is associated with disease progression in HIV-1-infected patients [23], [24], and likely influences the antiviral immune responses [23]–[25].

Cytotoxic T lymphocyte antigen 4 (CTLA-4) is overexpressed on activated T cells and inhibits IL-2 production and cell cycle progression by binding to its ligands B7-1 (CD80) and B7-2 (CD86) [26], [27]. CTLA-4 is also constitutively expressed on regulatory CD4+ T cells (Treg) and mediates T cell suppression [27]. Treg inhibits T cells via direct cell-cell interaction or indirectly by an antigen-presenting cell–mediated mechanism [28]. High CTLA-4 expression correlates with markers of HIV disease progression [29]–[31]. CTLA-4 is upregulated on HIV-specific CD4+, but not CD8+, T cells and in vitro blockade of CTLA-4 augments HIV-specific CD4+ T cell functions [29]. Upregulation of CTLA-4 also increases CCR5 expression and enhances susceptibility of CD4+ T cells to HIV infection [32]. The frequency of CTLA-4 positive Treg is increased in patients with chronic HIV-1 infection and is suspected to play a critical immunomodulatory role leading HIV-associated immune dysfunction [33]. In this study, we explore the relationship between CTLA-4 positive CD4+ T cells and the production of TGF-β and IL-10 by HIV-specific CD8+ T cells.

## Materials and Methods

### Study Subjects and Samples

HIV positive volunteers (n = 37) were recruited from the “The Research in Access to Care in the Homeless (REACH)” cohort in San Francisco as previously described [2], [34]. Demographic information and CD4+ T cell count was obtained at the time of enrollment and blood draw. Institutional Review Board approvals were obtained from the California Department of Public Health and UCSF Committee on Human Research, and all study participants gave written informed consent. None of the study participants have received antiretroviral therapy (ART) for at least 6 months. HIV RNA level was determined from plasma using the Roche Amplicor 1.5 (Roche, Branchburg, New Jersey), as per manufacturer's recommendations. Peripheral blood mononuclear cells (PBMC) were separated and cryopreserved in liquid nitrogen until assay time.

### Antigens

Peptides corresponding to the clade B consensus sequences of HIV-1 for Gag and Nef were synthesized as 15 amino acids (a.a.) overlapping by 11 a.a. (Mitochor Mimotopes,Victoria, Australia). Synthetic peptides for Gag (total  = 123), and Nef (total  = 49), used for all T cell assays were pooled into one single pool of peptides with final concentration of 1 µg/ml per peptide [35].

### Flow-Based Intracellular Cytokine Staining

Detection of HIV-specific TGF-β, IL-10, and IFN-γ production was performed as previously described [36]. PBMC (1×10^6^) were incubated with Gag or Nef peptide pools for 2 hours at 37°C in 5% CO_2_ in the presence of one of these following antibodies: anti-TGF-β R II (2 µg/ml, R&D systems, Minneapolis MN), anti-CTLA-4 (5 µg/ml, BD Phamingen), or the respective isotype control for 12–14 hours at 37°C in 5% CO_2_ in the presence of co-stimulatory anti-CD49d (1 µg/ml, Becton-Dickinson) and Golgi stop (BD Pharmigen). Phorbol myristate acetate (PMA, 50 ng/ml) and ionomycin (1 µg/ml; Sigma-Aldrich, St. Louis, MO), Lypopolysacccharide (LPS, 1 ng/ml; Sigma-Aldrich), and phytohaemagglutinin (PHA, 10 µg/ml; Sigma-Aldrich) were used as positive controls for TGF-β, IL-10 and IFN-γ production respectively. Media alone without antigen stimulation was used as negative control. All samples were stained with an amine reactive viability dye as a dead cell exclusion marker (Molecular probes, Eugene, OR) [37]. In some experiments depletion of CD4+ T cells was performed using the MACS CD4 depletion kit following the manufacturer's protocol (Miltenyi Biotec, Germany). Cells were then stained with specific combinations of the following antibodies: CD27 FITC, CD4 PerCP CY5.5, CD127 Alexa Fluor 647, CD8 PE CY7, CD3 AmCyan (BD Pharmigen). PBMC were permeabilized and stained with different combinations of the following antibodies: CTLA-4 APC, IL-10 PE, IL-10 APC, IFN-γ FITC (BD Pharmigen), TGFβ PE (Biotest Diagnostics, Denville, NJ), and analyzed by flow cytometry. A minimum of 30,000 CD3+ T cells per sample were acquired using a 6-color flow cytometer (LSRII, BD Biosciences) and analysis was performed by FLOWJO software (TreeStar, San Carlos, CA). Results were expressed as: Percent TGF-β, IL-10, or IFN-γ positive CD8+, T cells (Percent positive  =  % antigen-specific - % negative control). Responses greater than or equal to 0.1% and 2 times the background were considered positive. The extent of CTLA-4, CD27, and CD127 expression was also assessed. All volunteers demonstrated significant TGF-β, IL-10, and IFN-γ production following PMA/Ionomycin, LPS, and PHA stimulation respectively. Background expression was <0.1 percent.

### Flow-Based FOXP3 Staining Assay

PBMC were stained for FOXP3 expression following the manufacturer's protocol (eBioscience, San Diego, CA) with some modifications [2]. Briefly, PBMC (1×10^6^) were incubated with HIV peptide pools for 12–14 hrs as described above for intracellular cytokine staining. Cells were then stained with an amine reactive viability dye as a dead cell exclusion marker (Molecular probes) [37], and the following antibodies: FOXP3 FITC (eBioscience), TGF-β PE, CD4 PerCP Cy5.5, CD8 PE CY7, CD25 allophycocyanin, and CD3 AmCyan (BD Pharmigen). Analysis was performed by flow cytometry as described above. The percentage of TGF-β positive CD8+ T cells was determined and the extent of FOXP3 and CD25 expression was also assessed. Results were expressed as the fraction of TGF-β positive cells that expressed FOXP3 or CD25 over the total number of TGF-β positive cells (equivalent to 100%).

### Statistical Analysis

Groups were compared using the Mann-Whitney *U* test or the paired *t* test. Analysis was performed with PRISM software version 4.02 (Graph-Pad). Statistical significance was defined as *p*<0.05.

## Results

### TGF-β, IL-10, and IFN-γ Producing HIV-Specific CD8+ T Cells Are Distinct Populations

**Figure 1:**
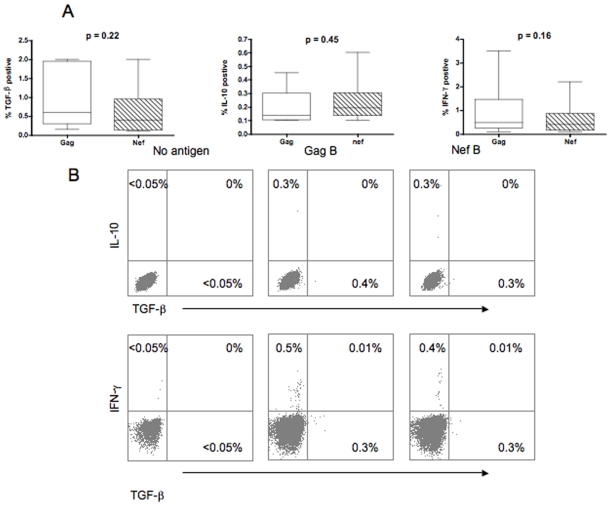
TGF-β positive, IL-10 positive, and IFN-γ positive HIV-specific CD8+ T cell populations are distinct. PBMC were stimulated with HIV peptides then stained with anti-IFN-γ FITC, anti-TGF-β PE, anti-CD3 AmCyan, anti-CD4 PerCP Cy5.5, and anti-CD8 PE Cy7, anti-IL-10 APC, and analyzed by flow cytometry. Samples were first gated on the CD3+/CD8+ lymphocyte population then the percent of TGF-β, IFN-γ, and IL-10 positive CD8+ T cells were determined. (A) Data from individuals with significant cytokine expression and analysis were performed by Mann-Whitney *U* test. (B) Representative plots of the number of HIV-specific CD8+ T cells expressing TGF-β, IFN-γ, and IL-10 after subtraction of the back ground values.

Specific populations of HIV-specific CD8+ T cells have been shown to express high TGF-β and IL-10 levels [18], [25], [38]. We first measured the frequency of Gag and Nef-specific TGF-β positive and IL-10 positive CD8+ T cells in 37 HIV-1 infected volunteers (Fig. 1A). IFN-γ production by Gag- and Nef-specific CD8+ T cells was determined concurrently. Volunteers had a median age of 45 years (range, 31–61), CD4+ T cell count of 168 cells/mm^3^ (range, 19–233), and median HIV plasma RNA of 132200 copies/ml (range, 19000–780000). Representative plots of the frequency of the HIV-specific TGF-β positive, IL-10 positive, and IFN-γ positive CD8+ T cells are shown in Fig. 1B. No overlap between HIV-specific CD8+ T cells producing TGF-β, IL-10 or IFN-γ was observed. All volunteers demonstrated significant Gag- (median  = 0.48%; range  = 0.1%−3.5%) and Nef- (median  = 0.4%; range  = 0.1%−2.2%) specific IFN-γ positive CD8+ T cell responses. Fourteen and fifteen volunteers demonstrated significant Gag-, and Nef-, specific TGF-β positive CD8+ T cell responses respectively (median  = 0.6%; range  = 0.15%−2.0%, and median  = 0.4%; range  = 0.1%−2.0% for Gag and Nef, respectively). Sixteen volunteers demonstrated significant Gag- (median  = 0.13%; range  = 0.1%−0.45%) and Nef- (median  = 0.19%; range  = 0.1%−0.6%) specific IL-10 positive CD8+ T cell responses. Differences in the frequencies of Gag- and Nef-specific TGF-β (or IL-10) positive CD8+ T cell responses was not significant (p = 0.22 and p = 0.45 for TGF-β and IL-10 respectively). We have previously demonstrated that HIV-specific IL-10 positive CD8+ T cells are increased in advanced HIV disease and are positively correlated with plasma HIV RNA [38]. No correlation was found between the frequency of the HIV-specific TGF-β positive CD8+ T cells and the CD4 count or plasma HIV RNA (for Gag: p = 0.07 and p = 0.1, for Nef: p = 0.68 and p = 0.13, for CD4 count or plasma HIV RNA respectively; data not shown). However, the relatively small number of volunteers in the study limited the full examination of this relationship.

### TGF-β Receptor II Blockade Increases IFN-γ Expression by HIV-Specific CD8+ T Cells

We have previously described the presence of suppressor HIV-specific IL-10 positive CD8+ T cells [2], [38]. We hypothesized that HIV-specific TGF-β positive CD8+ T cells also exert immunosuppressive functions [15]–[18], [23], [24]. To determine the effect of TGF-β on the HIV-specific effector functions (as measured by IFN-γ production), PBMC from three HIV-positive volunteers with evidence of Gag- and Nef-specific TGF-β positive CD8+ T cells were co-cultured in the presence of anti-TGF-β receptor II Ab (or isotype control). Blocking of the TGF-β receptor II led to increased Gag- and Nef-specific IFN-γ positive CD8+ T cell responses (data not shown). These results suggest that TGF-β production by CD8+ T cells inhibits HIV-specific effector functions.

### Analysis of the Immunophenotypic Profile of HIV-Specific TGF-β Positive CD8+ T Cells

**Figure 2:**
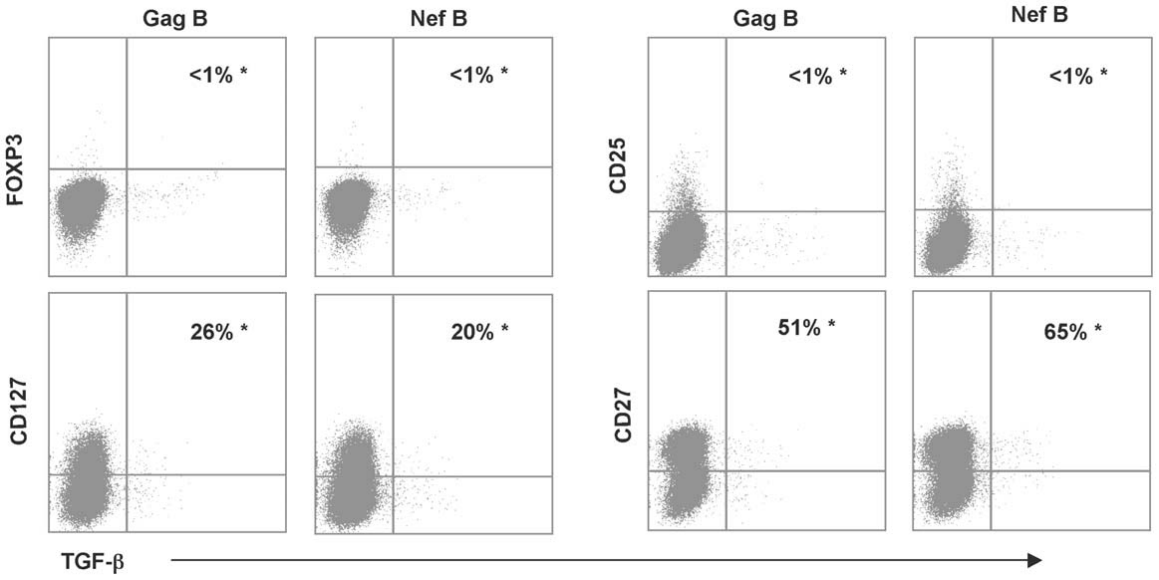
Analysis of regulatory surface markers expression by HIV-specific TGF-β positive CD8+ T cells. PBMC were stimulated with HIV peptides, then stained for various memory and regulatory markers and the percentage of TGF-β positive CD8+ T cells was determined by flow cytometry. Samples were first gated on the CD3+/CD8+ lymphocyte population and then the percentages of TGF-β positive cells were determined and the extent of FOXP3, CD127, CD25, and CD27 expression was also examined. Gating was performed using the fluorescence-minus-one (FMO) control for each marker. Representative plots of the phenotype of the HIV-specific CD8+ T cells expressing TGF-β. The values marked with an asterisk represent the fraction of TGF-β positive cells that express FOXP3, CD127, CD25, or CD27 over the total number of TGF-β positive cells (equivalent to 100%).

We have previously shown that the suppressor HIV-specific IL-10 positive CD8+ T cells were FOXP3 and CD25 negative [2], and displayed a heterogenous memory phenotype that was distinct from the HIV-specific effector CD8+ T cell population [38]. In this study, we examined the immunophenotypic profile of the HIV-specific TGF-β positive CD8+ T cells. PBMC were stimulated with HIV peptides and the extent of FOXP3, CD25, CD127, and CD27 expression by the HIV-specific TGF-β positive CD8+ T cells was assessed. Representative plots are shown in Fig. 2. HIV-specific TGF-β positive CD8+ T cells were FOXP3 negative and CD25 negative. Similar to the IL-10 positive CD8+ Ts, Gag-specific TGF-β positive CD8+ T cells displayed heterogeneous expression of CD27 and CD127. In contrast, HIV-specific IFN-γ positive CD8+ T cells were CD27 and CD127 negative (data not shown). These results indicate that the HIV-specific TGF-β positive CD8+ T cells are different from the population of suppressor CD8+ T cells in HIV negative individuals [1], [4], [11], [12], [39].

### CTLA-4 Blockade Decreases TGF-β and IL-10 Expression by HIV-Specific CD8+ T Cells

**Figure 3:**
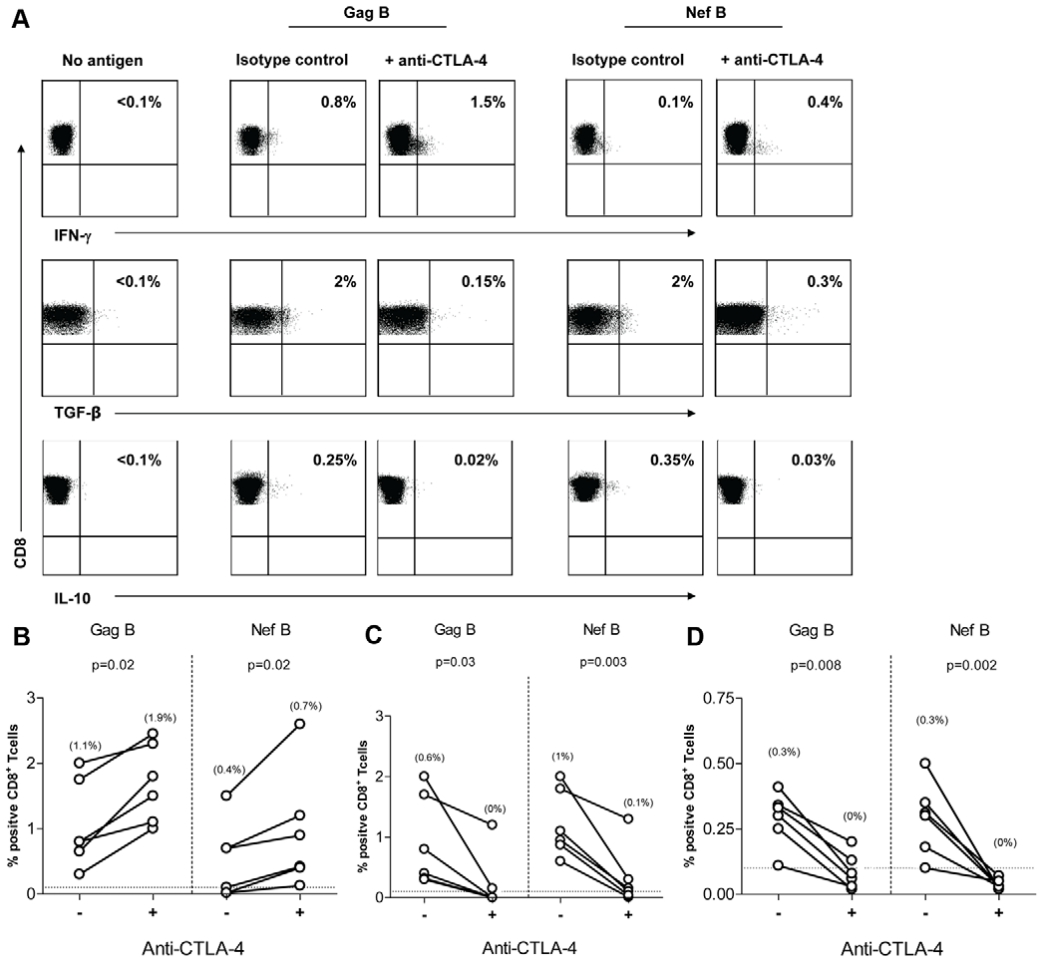
CTLA-4 blockade decreases TGF-β and IL-10 expression by HIV-specific CD8+ T Cells. PBMC (n = 6) were stimulated with HIV peptides in the presence of anti-CTLA4 (or isotype control), then stained with anti-IFN-γ FITC, anti-TGF-β PE, anti-IL-10 APC, anti-CD3 AmCyan, anti-CD4 PerCP CY5.5, anti-CD8 PE CY7, and analyzed by flow cytomerty. Samples were first gated on the CD3+/CD8+ lymphocyte population then the percent of TGF-β, IL-10, and IFN-γ positive cells were determined. Results were expressed as percent of HIV-specific CD8+ T cells expressing TGF-β, IL-10, or IFN-γ after subtraction of the back ground. (A) Representative plots of HIV-specific CD8+ T cells expressing TGF-β, IL-10, or IFN-γ in the presence or absence of anti-CTLA-4. (B-D) Dashed line represents the cutoff for significant TGF-β (B), IL-10 (C), and IFN-γ (D) expression. Percentages in between brackets are median values. The two dots joined by a line represent the values obtained from the same individual and analysis was performed by paired *t-*test.

In vitro blockade of CTLA-4 engagement augments HIV-specific CD4+ T cell proliferation, IL-2, and IFN-γ production [29]. We explored whether this inhibitory mechanism is involved in HIV-specific TGF-β and IL-10 CD8+ T cells. PBMC from 6 HIV-positive volunteers with demonstrated Gag- and Nef-specific TGF-β and IL-10 positive CD8+ T cell responses were incubated with anti-CTLA-4 Ab (or isotype control). Representative plots are shown in Fig. 3A. CTLA-4 blockade resulted in a significant decrease in the frequency of HIV-specific TGF-β (Fig. 3C) and IL-10 (Fig. 3D) positive CD8+ T cell responses. In contrast, blockade of CTLA-4 led to a significant increase in the frequency of HIV-specific IFN-γ positive CD8+ T cell responses (Fig. 3B). These results suggest that engagement of CTLA-4 regulates TGF-β and IL-10 production by HIV-specific CD8+ T cells.

### HIV-Specific TGF-β and IL-10 Positive CD8+ T Cells Are CTLA-4 Negative

**Figure 4:**
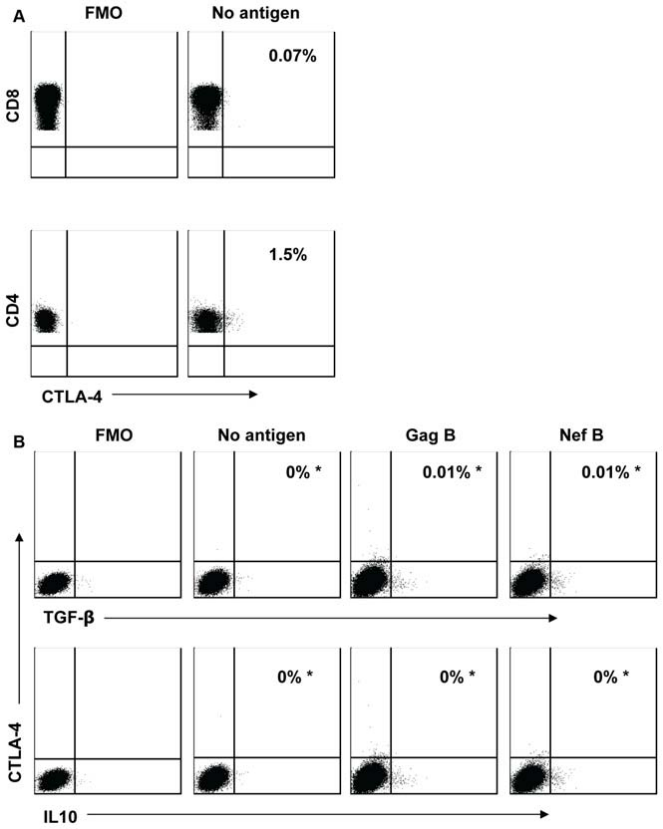
HIV-specific TGF-β and IL-10 positive CD8+ T cells are CTLA-4 negative. PBMC were stimulated with HIV peptides then stained with anti-TGF-β PE (or IL-10 PE), anti-CD3 AmCyan, anti-CD4 PerCP Cy5.5, anti-CD8 PE Cy7, anti-CTLA-4 APC, and analyzed by flow cytometry. Gating on the CTLA-4 positive cells was performed using the fluorescence-minus-one (FMO) control for CTLA-4. (A) Representative plots of samples that were first gated on the CD3+/CD4+ and CD3+/CD8+ lymphocyte population and then the percentages of CTLA-4 positive cells were determined. (B) Representative plots of samples that were first gated on the CD3+/CD8+ lymphocyte population and then the percent of TGF-β and IL-10 positive cells that express CTLA-4 was determined after subtraction of the back ground values. The values marked with an asterisk represent the fraction of TGF-β (or IL-10) positive cells that express CTLA-4 over the total number of TGF-β (or IL-10) positive cells (equivalent to 100%). Plots are from three independent experiments yielding similar results.

CTLA-4 is upregulated in HIV-specific CD4+ T cells [29], particularly in patients with chronic HIV-1 infection [33]. We next determined whether CTLA-4 is also expressed on TGF-β and IL-10 positive CD8+ T cells. Representative plots are shown in Fig. 4. We found no evidence of CTLA-4 expression on CD8+ T cells, including TGF-β and IL-10 positive CD8+ T cells (Fig. 4B) as well as on CD8+ IFN+ T cells (data not shown). This is in contrast to the high CTLA-4 expression detected on CD4+ T cells (median  = 2.8%; range  = 1.3%−7.0%; Fig. 4A). The lack of CTLA-4 expression on the CD8+ T cells suggests that the effect of CTLA-4 blockade on the TGF-β and IL-10 positive CD8+ T cells is mediated by an indirect mechanism.

**Figure 5:**
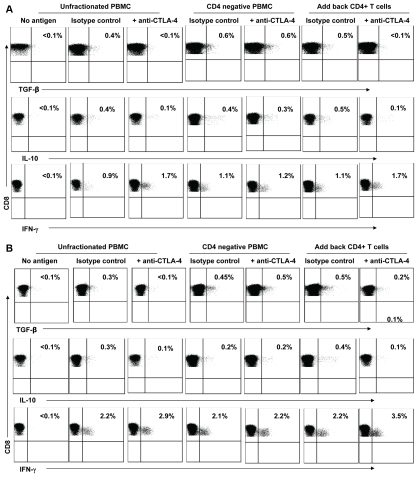
Removal of CD4+ T cells abrogates the effects of CTLA-4 blockade on HIV-specific CD8+ T Cells. PBMC (or CD4 negative PBMC) were stimulated with HIV peptides in the presence of anti-CTLA4 (or isotype control), then stained with anti-IFN-γ FITC, anti-TGF-β PE, IL-10 APC, anti-CD3 Am Cyan, anti-CD4 PerCP CY5.5, anti-CD8 PE CY7, and analyzed by flow cytomerty. Samples were first gated on the CD3+/CD8+ lymphocyte population then the percent of TGF-β, IL-10, and IFN-γ positive cells were determined. Results were expressed as percent of HIV-specific CD8+ T cells expressing TGF-β, IL-10, or IFN-γ after subtraction of the back ground. Representative plots of (A) Gag and (B) Nef-specific CD8+ T cells expressing TGF-β, IL-10, or IFN-γ in the presence or absence of anti-CTLA-4. Data plots shown are representative of three volunteers examined in three independent experiments yielding similar results.

We next attempted to further elucidate the role of CTLA-4 on TGF-β and IL-10 production by HIV-specific CD8+ T cells. We depleted CD4+ T cells in order to examine the effect of CTLA-4 expression by these CD4+ T cells on TGF-β and IL-10 production. Removal of CD4+ T cells fully abrogated the effect of CTLA-4 blockade on TGF-β, IL-10, and IFN-γ production by HIV-specific CD8+ T cells that we described above (Fig. 5). Adding back the autologous CD4+ T cells fully restored the effect of CTLA-4 blockade on TGF-β-, IL-10-, and IFN-γ-positive HIV-specific CD8+ T cells. While a relatively small number of volunteers were examined in the study, these results strongly support the role of CTLA-4 positive CD4+ T cells in the regulation of TGF-β, IL-10, and IFN-γ production by HIV-specific CD8+ T cells.

## Discussion

The mechanisms leading to the progressive loss of immune function and eventual failure of T cell responses in chronic HIV-1 infection remain unresolved. One hypothesis is that effector T cells succumb to an immunosuppressive environment. Multiple regulatory pathways are functionally altered and different CD4+ Treg subpopulations are induced in different stages of HIV infection [reviewed in [40]]. Similarly, distinct subpopulations of CD8+ Ts may also contribute to the immune dysregulation observed in HIV infection [1], [2], [4], [38]. Suppressor CD8+ T cells produce IL-10 and TGF-β, and mediate non-antigen specific suppression of T cell responses [41]–[43].

Our current study focuses on the mechanisms of suppression utilized by CD8+ Ts in inhibiting HIV-specific CD8+ T cell responses. The inhibition of HIV-specific T-cell responses by CD8+ Ts has been attributed to TGF-β and IL-10 production [18], [38]. We have previously described the presence of IL-10 positive CD8+ Ts that inhibited HIV-specific cytolysis and IL-2 production by direct cell-cell interaction [2], and indirectly by releasing IL-10 [38]. The presence of these suppressor IL-10 positive CD8+ T cells was associated with increased expression of the inhibitory receptor programmed cell death-1(PD-1) on exhausted HIV-specific effector CD8+ T cells [36]. However, blockade of the PD-1/PDL-1 pathway did not prevent suppression, suggesting that PD-1 engagement is not a mechanism utilized by the IL-10 positive CD8+ T cells. We now describe an HIV-specific TGF-β positive CD8+ T cell population that is distinct from IL-10 positive CD8+ Ts. Blocking the binding of TGF-β to its receptor resulted in increased IFN-γ expression by HIV-specific CD8+ T cells. We postulate that these TGF-β positive CD8+ T cells exert an inhibitory effect on HIV-specific effector function in vitro. Nevertheless, the suppressive impact of these TGF-β CD8+ T cells in vivo remains unknown, and direct cell-cell contact may be required for the these TGF-β positive CD8+ T cells to exert their maximum inhibitory effect. The intriguing role by which distinct antigen-specificity of these TGF-β positive CD8+ T cells contributes to the level of suppression is remains to be explored [18].

We have previously shown that IL-10 positive CD8+ Ts are observed in late HIV disease [38]. The induction and detection of TGF-β positive CD8+ T cells in the course of HIV disease is still unknown but may develop earlier in HIV disease [44]. Because TGF-β is a key regulator in the suppressive signaling pathways implicated in immune regulation, signaling through TGF-β receptor may promote differential and temporal effect on T cells in different stages of HIV disease. We have shown previously that the concomitant presence of Gag- and Nef-specific IL-10 positive CD8+ Ts augmented the in vitro suppressive effect on effector CD8+ T cells [38]. We postulate that the concomitant presence of HIV-specific IL-10 positive and TGF-β positive CD8+ T cells will promote additive suppression of effector CD8+ T cell responses.

In this study we describe the presence of TGF-β positive CD8+ T cells with multiple antigen specificities that displayed an immunophenotype profile that is similar to our previously described IL-10 positive CD8+ Ts [2]. These TGF-β positive CD8+ T cells do not display the immunophenotypic patterns traditionally attributed to regulatory T cells. FOXP3 and CD25 surface expressions are the hallmark for identifying CD8+ Ts in diseases other than HIV infection [1], [3], [4], [13], [14], [45]–[47]. The lack of CD127 expression on FOXP3 positive CD4+ T cells is used to distinguish between regulatory and effector T cells [48], [49]. However, FOXP3 negative and CD25 negative CD8+ Ts have also been described [2]. The mechanisms leading to the induction of these TGF-β positive CD8+ T cells likely share similar pathways with other regulatory T cells. One key question is whether CD4+Treg are required for the generation and suppressive function of CD8+Ts.

Inhibitory functions of regulatory T cells are mediated by direct binding of CTLA-4 and by cytokine production [28], [50], [51]. Surprisingly, engagement of CTLA-4 prevented the production of suppressive cytokines by the CTLA-4 negative HIV-specific CD8+ T cells. We propose a model by which CTLA-4 positive CD4+ T cells indirectly influence the inhibitory function of HIV-specific CD8+ Ts by CTLA-4 engagement. The abrogation of anti-CTLA-4 blockade in the absence of CD4+ T cells supports our hypothesis. CTLA-4 is upregulated on HIV-specific CD4+ T cells in advanced disease [29]-[31], and CTLA-4 is also constitutively expressed on regulatory CD4+ Treg [27]. Clearly, the presence of CD4+ Treg in HIV infection adds to the complexity of immune regulation and suppression. The influence of CTLA-4 expression by populations other than CD4+ T cells on the effectiveness of CD8+ suppressor T cells remains to be determined.

In summary, our study described an HIV-specific CD8+ T cell population that mediated in vitro suppression of HIV-specific effector function through the production of TGF-β. These TGF-β positive CD8+ T cells most likely confer regulatory properties. Nevertheless, the relative contribution of these TGF-β positive CD8+ T cells to the general immune dysfunction observed in HIV infection is currently unknown. The impact of HIV-specific CD8+ Ts remains an intriguing new research area that offers new direction into the consequence of HIV immunopathogenesis. Further studies should focus on the pathways by which complex immune regulatory mechanisms modulate the adaptive, antiviral immune response. The ability to manipulate and regulate effective antiviral immunity provides an approach toward decreasing the number of infected cells and improving disease outcome.
